# Can empirical hypertonic saline or sodium bicarbonate treatment prevent the development of cardiotoxicity during serious amitriptyline poisoning? Experimental research

**DOI:** 10.5830/CVJA-2015-014

**Published:** 2015

**Authors:** Muhammet Sukru Paksu, Halit Zengin, Adem Uzun, Fatih Ilkaya, Hasan Guzel, Sule Paksu, Durmus Ucar, Hasan Alacam, Latif Duran, Naci Murat, Ahmet Guzel

**Affiliations:** Paediatric Intensive Care Unit, Faculty of Medicine, Ondokuz Mayis University, Samsun, Turkey; Department of Cardiology, Faculty of Medicine, Ondokuz Mayis University, Samsun, Turkey; Department of Cardiology, Faculty of Medicine, Ondokuz Mayis University, Samsun, Turkey; Department of Pharmacology, Faculty of Medicine, Ondokuz Mayis University, Samsun, Turkey; Department of Pharmacology, Faculty of Medicine, Ondokuz Mayis University, Samsun, Turkey; Department of Paediatrics, Faculty of Medicine, Ondokuz Mayis University, Samsun, Turkey; Department of Physiology, Faculty of Medicine, Ondokuz Mayis University, Samsun, Turkey; Department of Biochemistry, Faculty of Medicine, Ondokuz Mayis University, Samsun, Turkey; Department of Emergency Medicine, Faculty of Medicine, Ondokuz Mayis University, Samsun, Turkey; Department of Industrial Engineering, Faculty of Engineering, Ondokuz Mayis University, Samsun, Turkey; Department of Paediatric Emergency, Faculty of Medicine, Ondokuz Mayis University, Samsun, Turkey

**Keywords:** amitriptyline, poisoning, cardiotoxicity, sodium bicarbonate, hypertonic saline

## Abstract

**Objective:**

The aim of this experimental study was to investigate whether hypertonic saline or sodium bicarbonate administration prevented the development of cardiotoxicity in rats that received toxic doses of amitriptyline.

**Method:**

Thirty-six Sprague Dawley rats were used in the study. The animals were divided into six groups. Group 1 received toxic doses of i.p. amitriptyline. Groups 2 and 3 toxic doses of i.p. amitriptyline, plus i.v. sodium bicarbonate and i.v. hypertonic saline, respectively. Group 4 received only i.v. sodium bicarbonate, group 5 received only i.v. hypertonic saline, and group 6 was the control. Electrocardiography was recorded in all rats for a maximum of 60 minutes. Blood samples were obtained to measure the serum levels of sodium and ionised calcium.

**Results:**

The survival time was shorter in group 1. In this group, the animals’ heart rates also decreased over time, and their QRS and QTc intervals were significantly prolonged. Groups 2 and 3 showed less severe changes in their ECGs and the rats survived for a longer period. The effects of sodium bicarbonate or hypertonic saline treatments on reducing the development of cardiotoxicity were similar. The serum sodium levels decreased in all the amitriptyline-applied groups. Reduction of serum sodium level was most pronounced in group 1.

**Conclusion:**

Empirical treatment with sodium bicarbonate or hypertonic saline can reduce the development of cardiotoxicity during amitriptyline intoxication. As hypertonic saline has no adverse effects on drug elimination, it should be considered as an alternative to sodium bicarbonate therapy.

## Abstract

Tricyclic antidepressant (TCA) drugs are commonly used to treat many neuropsychiatric diseases.^1^ Amitriptyline is the most commonly prescribed antidepressant, and it is a frequent cause of toxicity in drug overdoses. TCA overdose primarily affects the neurological, cardiovascular and respiratory systems.^1^,^2^

Blockage of the rapid sodium channels is responsible for drug-induced cardiotoxicity, which clinically manifests as PR, QT and QRS prolongation, ventricular or supraventricular arrhythmias, hypotension and heart failure.^1^,^3^ Sodium bicarbonate (NaHCO_3_) administration is the most widely accepted treatment to reduce amitriptyline-induced cardiotoxicity.^2^,^4^,^5^ However, at an alkaline pH, the volume distribution of amitriptyline expands, and the elimination time is longer. Therefore, NaHCO_3_ treatment is suggested only in the presence of cardiac findings.^6^ Hypertonic saline (HS) administration has been shown to be useful, particularly when cardiotoxicity is accompanied by hypotension.^7^-^9^

There is always a need for a medication to prevent cardiotoxicity that will save patients’ lives, especially when toxic doses have been ingested. Although studies have compared the efficacy of HS and NaHCO_3_ treatments in patients with cardiotoxicity, the role of these therapies to prevent or reduce cardiotoxicity in patients who may potentially develop severe toxicity has not been investigated. The aim of this experimental study was to compare the effect of early administration of HS or NaHCO_3_ on preventing cardiotoxicity in rats that had received toxic doses of amitriptyline.

## Methods

The experiments were performed on adult female Sprague Dawley rats weighing 250 to 300 g that were obtained from the Ondokuz Mayis University vivarium. The rats were kept in a vivarium maintained at 22 ± 1°C with a 12-hour alternating light–dark cycle. All the experiments were approved by the Institutional Animal Care and Use Committee of Ondokuz Mayis University, and adhered to the guidelines of the Committee on Human/Animal Experimentation (institutional or regional), and the Helsinki Declaration of 1975, as amended in 1983.

Amitriptyline was obtained from Sigma-Aldrich Chemical Co (St Louis, Missouri, USA). It was dissolved in distilled water at a concentration of 50 mg/4 ml. HS solution (3% sodium chloride, sodium 512 mEq/l) and NaHCO3 8.4% (sodium 1 mEq/ml) were used.

The animals were randomly divided into six groups, with each group containing six rats. They were anesthetised (100 mg/kg ketamine and 0.75 mg/kg chlorpromazine i.p.) and then prepared for monitoring of electrocardiogram (ECG) parameters. Their survival time was recorded by a data-acquisition system (ML870/P, PowerLab 8/30, AD Instruments).

Amitriptyline was administered at a dose 50 mg/kg i.p. to induce toxicity. The HS was administrated at a dose rate of 6 ml/kg, and the NaHCO3 was administrated at a dose rate of 3 mEq/kg. Both were administered via i.v. infusion and applied simultaneously with amitriptyline over a period of five minutes. To administer the dosage, i.v. cannulas were inserted into the tail of the rats. The toxic doses and treatments given to the different groups are shown in Table 1.

**Table 1:** Design of study and groups and chemicals used

*Group*	*Drugs*
1	Only amitriptyline (50 mg/kg i.p.)
2	Amitriptyline (50 mg/kg i.p.) + 3 mEq/kg NaHCO_3_ (diluted with normal saline of 1:1 ratio) during the five minutes (once)
3	Amitriptyline (50 mg/kg i.p.) + 6 ml/kg hypertonic saline during the five minutes (once)
4	Only 6 ml/kg hypertonic saline during the five minutes (once)
5	Only 3 mEq/kg NaHCO_3_ (diluted with normal saline of 1:1 ratio) during the five minutes (once)
6	Control group (none of the drugs or treatment)

ECGs were recorded on each rat for 60 minutes after the administration of the respective protocols. All records were evaluated by a cardiologist. On the ECG records, the R–R distance, height of the QRS and duration of the QT were measured. The R–R is the interval from the onset of one QRS complex to the onset of the next QRS complex, measured in seconds. The heart rate and the corrected QT (QTc) interval were calculated according to the R–R distance and the duration of the QT. Heart rate was calculated by dividing the R–R interval by 60 (heart rate = 60/R–R interval).

The QRS duration was measured from the beginning of the Q wave to the end of the S wave. The QT interval was measured from the onset of the QRS complex to the end of the T wave, defined as the return to the TP isoelectric line. The QT interval was defined as the average of the QT intervals of three consecutive beats in each of the ECG leads. The QT intervals were also corrected for heart rate using Bazett’s formula. The QTc is equal to the QT interval in seconds divided by the square root of the preceding R–R interval in seconds. A decrease in the heart rate below 100 beats/minutes or the presence of asystole during recording was accepted as the exodus.

To measure serum levels of sodium and ionised calcium, blood samples were obtained from the carotid arteries of the living rats 60 minutes after the administration of amitriptyline or other treatments, but immediately from the rats that had died.

## Statistical analysis

Statistical analyses were performed with IBM SPSS 21.0 (Chicago, IL, USA). The Kolmogorov–Smirnov test was used to evaluate the distribution of variables in relation to normal. Descriptive statistics were presented as the mean ± standard deviation. Statistical comparisons between all groups were performed with one-way ANOVA with a Tukey *post-hoc* test. Correlations between the quantitative data were analysed by the Pearson correlation test. The level of statistical significance was set at *p* < 0.05.

## Results

The characteristics of the rats in all groups were similar. In group 1, all the rats died in the first 25 minutes. Therefore, the statistical analyses with group 1 included data for only the first 25 minutes. The other inter-group statistical analyses included data obtained over 60 minutes.

The initial heart rate was similar among the groups. The heart rates of the rats in groups 1, 2 and 3 decreased in direct proportion to time, with the decrease more marked in group 1. The heart rates of the rats in groups 4, 5 and 6 did not show significant change over time.

**Figure 1.**
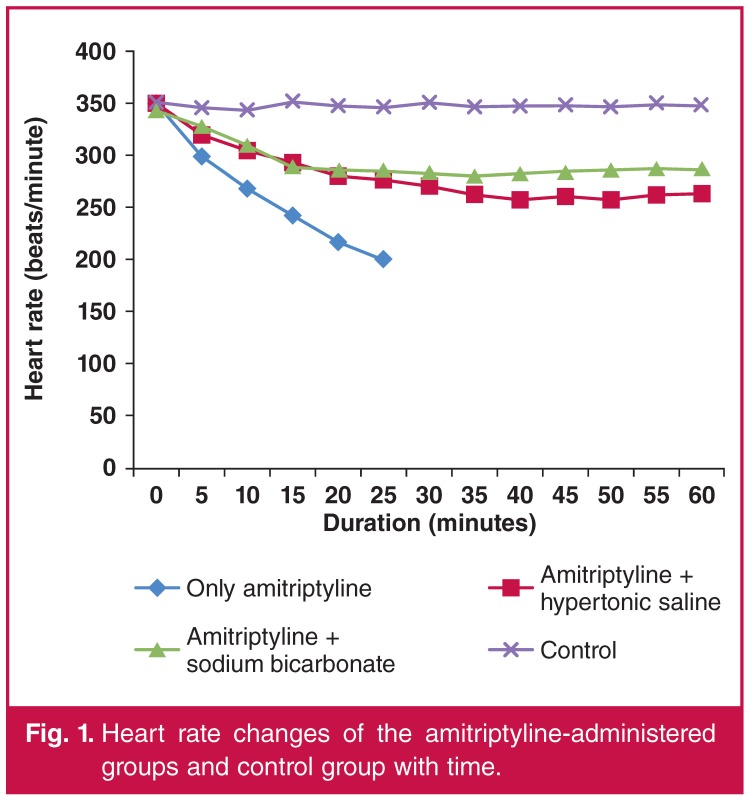
Heart rate changes of the amitriptyline-administered groups and control group with time.

Hypertonic saline or NaHCO_3_ administration, along with amitriptyline, ameliorated the reduction in the heart rates. There was no significant difference in the heart rates between the HS and NaHCO_3_ groups. Table 2 shows a comparison of the heart rates of the rats in each group in each time period. The changes in the heart rates in the amitriptyline-treated groups (groups 1, 2 and 3) and the control group are shown in Fig. 1.

**Table 2:** Heart rate changes with time according to group

*Group*	*Start*	*5th minute*	*10th minute*	*15th minute*	*20th minute*	*25th minute*
1	353 ± 21*	300 ± 37^c^	269 ± 31^c,d,e^	242 ± 31^a,c,d,e^	217 ± 28^a,b,c,d,e^	201 ± 28^a,b,c,d,e^
2	350 ± 17*	321 ± 34^g^	304 ± 34^g^	294 ± 34^a,g,h,i^	281 ± 33^a,g,h,i^	277 ± 32^a,g,h,i^
3	345 ± 19*	327 ± 25*	309 ± 30^j^	290 ± 32^b,j,k,l^	285 ± 32^b,j,k,l^	285 ± 31^b,j,k,l^
4	368 ± 26*	373 ± 27*	371 ± 23^c,g,j^	375 ± 23^c,j,g^	379 ± 23^c,g,j^	377 ± 22^c,g,j^
5	346 ± 14*	343 ± 21*	344 ± 18^d^	345 ± 22^d,h,k^	346 ± 23^d,h,k^	347 ± 28^d,h,k^
6	352 ± 34*	346 ± 23^c,g^	344 ± 23^e^	352 ± 27^e,i,l^	348 ± 23^e,i,l^	347 ± 26^e,i,l^
Mean	352 ± 23	335 ± 35	324 ± 42	316 ± 53	309 ± 60	312 ± 61
*p*-value	0.542	0.003	0.000	0.000	0.000	0.000

*The group with no difference from the others, *p* < 0.05 (^a^compared with groups 1 and 2, ^b^compared with groups 1 and 3, ^c^compared with groups 1 and 4, ^d^compared with groups 1 and 5, ^e^compared with groups 1 and 6, ^f^compared with groups 2 and 3, ^g^compared with groups 2 and 4, ^h^compared with groups 2 and 5, ^i^compared with groups 2 and 6, ^j^compared with groups 3 and 4, ^k^compared with groups 3 and 5, ^l^compared with groups 3 and 6).

**Figure 2.**
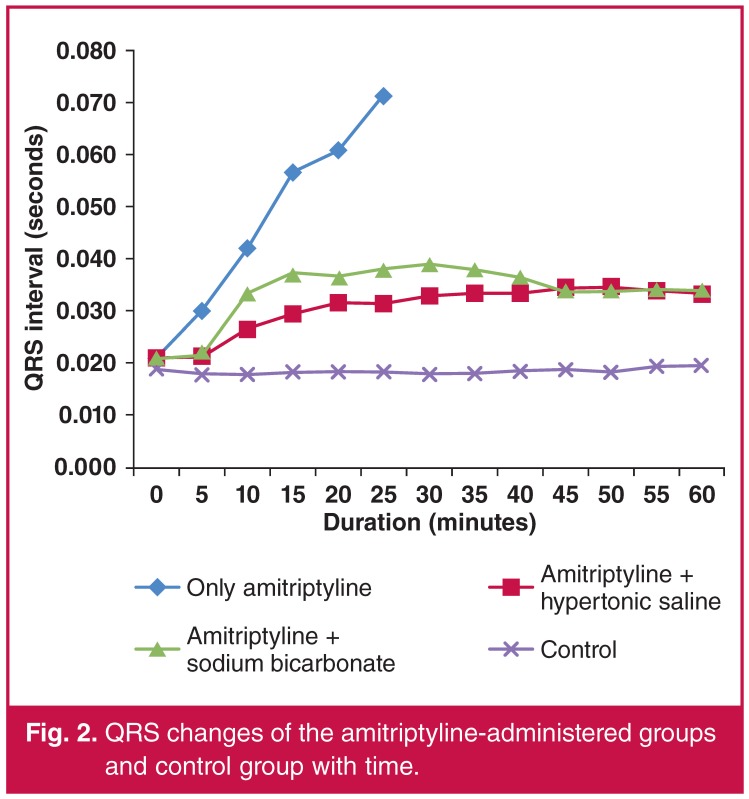
QRS changes of the amitriptyline-administered groups and control group with time.

The baseline QRS durations were similar among groups. The changes in the QRS durations in the groups that did not received amitriptyline (groups 4, 5 and 6) were non-significant, whereas the amitriptyline-administered groups (groups 1, 2 and 3) showed a statistically significant increase in the QRS duration. This increase was more marked in group 1. The increase in QRS duration was limited in the groups that received amitriptyline with HS or NaHCO3 (groups 2 and 3). There was no statistically significant difference between these groups in terms of increase in the duration of the QRS. A comparison of the QRS duration in each group in each time period is shown in Table 3, and a comparison of the change in QRS duration of the amitriptylinetreated groups (groups 1, 2 and 3) and control group is shown in Fig. 2.

**Table 3:** QRS changes with time according to group

*Group*	*Start*	*5th minute*	*10th minute*	*15th minute*	*20th minute*	*25th minute*
1	0.210 ± 0.001*	0.0303 ± 0.0015^c,e^	0.042 ± 0.01^a,c,d,e^	25th minute	0.061 ± 0.130^a,b,c,d,e^	0.072± 0.02^a,b,c,d,e^
2	0.208 ± 0.001*	0.022 ± 0.010*	0.027 ± 0.006^a^	0.030 ± 0.005^a^	0.032 ± 0.006^a,g,i^	0.032 ± 0.006^a,i^
3	0.208 ± 0.003*	0.022± 0.002*	0.034 ± 0.008^j,k,l^	0.038 ± 0.008 ^b,k,l^	0.037 ± 0.008^b,j,k, l^	0.038 ±0.009^b,j,k,l^
4	0.198 ± 0.001*	0.0190 ± 0.001^c^	0.020 ± 0.001^c,j^	0.020 ± 0.001^c^	0.019 ± 0.001^c,g,j^	0.020 ± 0.001^c,j^
5	0.202 ± 0.001*	0.020 ± 0.001*	0.020 ± 0.002^d,k^	0.020 ± 0.01d	0.021 ± 0.001^d,k^	0.020 ± 0.001^d,k^
6	0.195 ± 0.003*	0.0183 ± 0.02^e^	0.020 ± 0.002^d,k^	0.019 ± 0.002 ^e,k,l^	0.019 ± 0.001^e,i,l^	0.019 ± 0.001^e,i,l^
Mean	0.204 ± 0.002	0.022 ± 0.007	0.027 ± 0.01	0.030 ± 0.015	0.031 ± 0.016	0.031 ± 0.018
*p*-value	0.782	0.032	0.000	0.000	0.000	

*The group with no difference from the others, p < 0.05 (^a^compared with groups 1 and 2, ^b^compared with groups 1 and 3, ^c^compared with groups 1 and 4, ^d^compared with groups 1 and 5, ^e^compared with groups 1 and 6, ^f^compared with groups 2 and 3, ^g^compared with groups 2 and 4, ^h^compared with groups 2 and 5, ^i^compared with groups 2 and 6, ^j^compared with groups 3 and 4, ^k^compared with groups 3 and 5, ^l^compared with groups 3 and 6).

**Figure 3.**
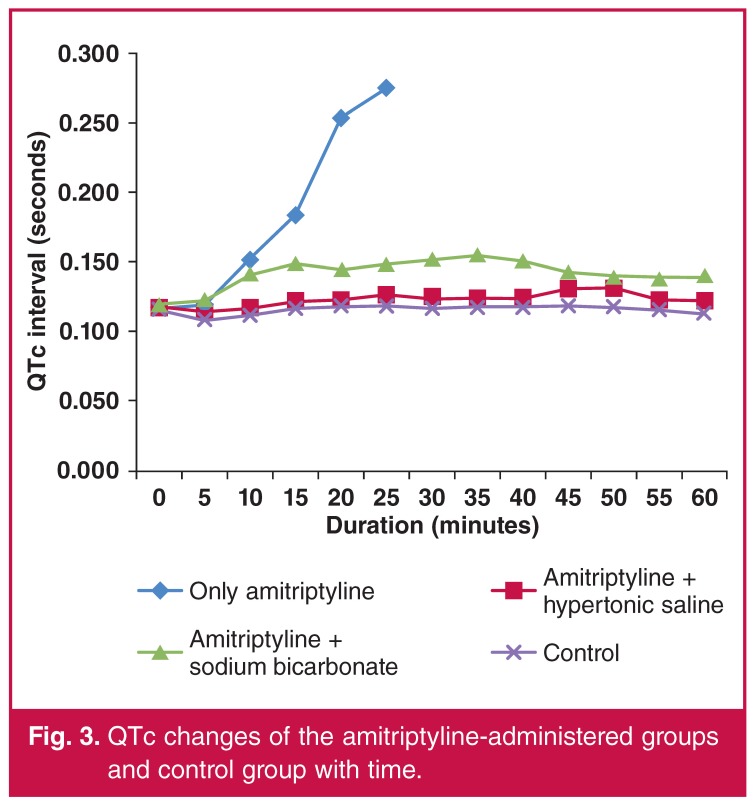
QTc changes of the amitriptyline-administered groups and control group with time.

The baseline QTc durations were similar among the groups. There was no significant change in the QTc durations in the groups that did not receive amitriptyline (groups 4, 5 and 6). The QTc duration increased in the amitriptyline-treated groups (groups 1, 2 and 3), but the increase was more marked in group 1. The increase in the QTc duration was reduced in the groups administered HS or NaHCO_3_ with amitriptyline. There was no statistically significant difference between the two groups (groups 2 and 3) in terms of QTc prolongation. A comparison of the QTc duration of the groups in each time period is shown in Table 4, and a comparison of the change in duration of the QTc in the amitriptyline-treated groups and the control group is shown in Fig. 3.

**Table 4:** QTc changes with time according to group

*Group*	*Start*	*5th minute*	*10th minute*	*15th minute*	*20th minute*	*25th minute*
1	0.119 ± 0.005*	0.115 ± 0.007*	0.154 ± 0.019a,c,d,e	0.187 ± 0.025a,b,c,d,e	0.255 ± 0.11a,b,c,d,e	0.277 ± 0.081a,b,c,d,e
2	0.118 ± 0.005*	0.115 ± 0.009*	0.119 ± 0.004a,f	0.124 ± 0.021a	0.124 ± 0.02a	0.129 ± 0.020a
3	0.119 ± 0.006*	0.115 ± 0.009*	0.143 ± 0.016f,j,k,l	0.150 ± 0.018 b,l	0.146 ± 0.02b	0.146 ± 0.02b
4	0.121 ± 0.006*	0.123 ± 0.007*	0.123 ± 0.007c,j	0.127 ± 0.07c	0.124 ± 0.005c	0.123 ± 0.008c
5	0.120 ± 0.002*	0.119 ± 0.005*	0.119 ± 0.004d,k	0.123 ± 0.005d	0.119 ± 0.004d	0.122 ± 0.005d
6	0.117 ± 0.006*	0.111 ± 0.009*	0.113 ± 0.007e,l	0.117 ± 0.007e,l	0.118 ± 0.001e	0.119 ± 0.008e
Mean	0.119 ± 0.006	0.111 ± 0.009*	0.128 ± 0.180	0.138 ± 0.03	0.148 ± 0.065	0.146 ± 0.057
p-value	0.654	0.0055	0.000	0.000	0.000	0.000

*The group with no difference from the others, *p* < 0.05 (^a^compared with groups 1 and 2, ^b^compared with groups 1 and 3, ^c^compared with groups 1 and 4, ^d^compared with groups 1 and 5, ^e^compared with groups 1 and 6, ^f^compared with groups 2 and 3, ^g^compared with groups 2 and 4, ^h^compared with groups 2 and 5, ^i^compared with groups 2 and 6, ^j^compared with groups 3 and 4, ^k^compared with groups 3 and 5, ^l^compared with groups 3 and 6).

Serum samples for sodium and ionised calcium analyses were taken from the surviving rats at the 60th minute and from the non-survivors immediately after death. In the groups that received amitriptyline (groups 1, 2 and 3), the serum sodium levels had decreased. This decline was most evident in group 1, which did not receive any sodium-containing treatment. Hyponatraemia was more pronounced in group 2 than group 3. No subject developed hypernatraemia.

The serum ionised calcium levels were higher in the groups that received amitriptyline and highest in the amitriptyline-only group (group 1). The distribution of the serum levels of ionised calcium and sodium in the groups is shown in Table 5. The serum sodium levels showed a strong positive correlation with heart rates, a strong negative correlation with QRS duration, and a significant moderately negative correlation with QTc duration. Serum ionised calcium levels exhibited a significant moderately negative correlation with heart rate and a significant moderately positive correlation with QRS and QTc duration (Table 6).

**Table 5:** Distribution of serum levels of ionised calcium and sodium according to group

*Group*	*Sodium*	*Calcium*
1	111.2 ± 4.3a	4.14 ± 0.4a
2	121.2 ± 6.2a	4.19 ± 0.4a
3	133.4 ± 10.9a	4.15 ± 1.0a
4	143.5 ± 3.4b	2.85 ± 0.6b
5	145.7 ± 4.7b	2.52 ± 0.7b
6	147.8 ± 4.6b	1.83 ± 0.7b
Mean	133.8 ± 14.9	3.28 ± 1.1
p-value	0.000	0.000

^a^No statically significant differences among groups 1, 2 and 3. ^b^No statically significant differences among groups 4, 5 and 6.

**Table 6:** Correlation between heart rate, QRS and QTc intervals and serum sodium and calcium levels

*Levels*		*Rate*	*QRS duration*	*QTc duration*
Sodium	p	0.000	0.000	0.000
	r	0.794**	–0.776**	–0.612**
Calcium	p	0.000	0.002	0.016
	r	–0.0620*	0.500**	0.399*

*Correlation significant at the 0.05 level. **Correlation significant at the 0.01 level.

## Discussion

The results of this experimental study suggest that administration of HS or NaHCO_3_ before toxicity occurs may reduce the development of cardiac toxicity in amitriptyline poisoning.

Overdose of TCAs, including amitriptyline, are a major causes of drug-related deaths all over the world.^10^ Amitriptyline poisoning primarily affects the cardiovascular and neurological systems.^1^,^11^,^12^ It causes toxicity by blocking the voltage-gated sodium channels, which facilitate the fast flow of sodium into the cells.^1^,^13^ Anticholinergic and α-adrenergic blockage also contribute to this.^1^,^3^,^13^,^14^ Blockage of cardiac sodium and potassium channels may result in cardiac conduction delay, dysrhythmia and hypotension due to myocardial depression.^1^,^2^,^4^,^14^ This process may appear on the ECG as prolonged PR, QRS and QT times, sinus tachycardia, and supraventricular and ventricular arrhythmias.^1^,^11^ The most important cause of death is persistent hypotension resulting from myocardial depression due to arrhythmias.^3^,^5^,^10^,^12^,^13^

The majority of patients who take toxic doses of amitriptyline enter a coma, but a minority develop life-threatening complications. Others often recover with supportive care, without subsequent problems.^15^,^16^ Despite defined scoring systems such as the Antidepressant Overdose Risk Assessment (ADORA),^17^ it is often difficult to distinguish these two groups of patients. In addition, the correlation between serum drug levels and clinical outcome is weak, and routine drug level analyses are not recommended.^18^,^19^

Various methods have been used to treat patients with severe cardiotoxicity due to amitriptyline overdose. These include serum alkalinisation with hypertonic NaHCO_3_^20^ or hyperventilation,^21^ inotropic agents,^4^,^22^ magnesium sulphate,^5^ anti-arrhythmic drugs,^2^,^14^ glucagon^12^ haemoperfusion,^23^,^24^ or lipid emulsion.^25^ Although many studies have compared these treatment methods, no treatment has been shown to prevent or reduce the toxicity in patients who may potentially develop severe toxicity.

Sodium bicarbonate is the first choice in the treatment of TCA-induced cardiotoxicity.^4^,^5^ There are several mechanism by which NaHCO_3_ treatment may confer beneficial effects. It may increase serum sodium levels and reduce cardiac arrhythmias resulting from the inhibition of sodium channels.^4^,^11^ It may reduce the alkaline pH of the serum concentration of the ionised fraction of the drug that is responsible for cardiotoxicity.^20^,^26^ In addition, it may give rise to volume expansion.^4^

Studies have shown that NaHCO_3_ treatment reduces the incidence of ventricular arrhythmias, prevents prolonged QRS and QT intervals, and improves hypotension.^11^,^20^ However, the disadvantages of this treatment include volume overload, hypokalaemia induced by alkalosis, and delay in the elimination of the drug in an alkaline pH.^4^ For this reason, it has been suggested that hypertonic NaHCO_3_ treatment should be used only in the presence of severe cardiac findings.^6^

Previous studies have frequently reported an association between TCA poisoning and hyponatraemia.^24^,^27^-^29^ Possible causes of this situation are sodium loss due to vomiting or gastric lavage, treatment with hypotonic fluids, or, most importantly, inappropriate secretion of antidiuretic hormone due to critical illness.

Our clinical observations of patients with amitriptyline poisoning suggested that there might be a relationship between hyponatraemia and the degree of poisoning, particularly with regard to the presence of cardiac arrhythmias and seizures.^30^ Therefore, in this study, we aimed to investigate whether HS therapy initiated before the development of signs of systemic toxicity would reduce the development of cardiotoxicity.

Hypertonic saline treatment is widely used for many indications, especially in hypovolaemic and septic shock, hyponatraemic encephalopathy and increased intracranial pressure syndrome, and clinicians have a very high degree of experience in this field.^31^-^34^ Earlier experimental studies have demonstrated the effects of the administration of HS in severe TCA poisoning.^7^-^9^ The main advantages of HS therapy are that it is cheap and accessible, and it has therapeutic properties in hyponatraemic, hypovolaemic or hypotensive patients.

In this study, the rats administered toxic doses of amitriptyline developed severe cardiotoxicity that resulted in a prolonged QRS and QTc duration on ECG, slower heart rates and even death. The apparent relationship between the depth of hyponatraemia and the clinical outcome led us to believe that sodium may be a key player in the treatment.

In our study, the administration of HS or NaHCO_3_ in the early stage of poisoning seemed to delay and reduce the development of toxicity. The effectiveness of both treatments was found to be similar. Similar amounts of sodium (~ 3 mEq/kg) were given to both groups. The group administered amitriptyline with NaHCO_3_ had borderline hyponatraemia. The cause of the more significant hyponatraemia in the group treated with HS is not clear. Further studies with different concentrations of sodium-containing fluids are needed to evaluate this issue.

Interestingly, serum ionised calcium levels were higher in the groups that received amitriptyline than in the control group in our study. We did not study blood pH and other factors that affect calcium metabolism. Therefore, the pathophysiological basis of our findings is unclear. An additional limitation of this study is that we did not study factors affecting calcium metabolism and blood gases.

## Conclusion

Amitriptyline poisoning is a common occurrence. Although the majority of cases improve with supportive therapy, cardiac complications may be life threatening in some cases. Although prospective, controlled human studies are needed, the results of this preliminary study suggest that amitriptyline poisoning leads to hyponatraemia, and early HS or NaHCO3 treatment may reduce the development of cardiac toxicity. As HS treatment does not affect serum levels of ionised calcium and potassium or change the drug elimination time, it may be preferred to NaHCO_3_ therapy.
